# Maternal Dietary B-Complex Vitamin Pattern and Risk of Complex Congenital Heart Defects in a Mexican Population

**DOI:** 10.3390/nu18162693

**Published:** 2026-08-18

**Authors:** Jenny Vilchis-Gil, Aaron M. Gómez-Jiménez, María E. Santillán-Orgas, Begoña Segura-Stanford, Iñaki Navarro-Castellanos, Jacqueline Gomez-Lopez, Mari C Morán-Espinosa, Javier T. Granados-Riveron, Rocío Sanchez-Urbina

**Affiliations:** 1 Unidad de Investigación Epidemiológica en Endocrinología y Nutrición, Hospital Infantil de México Federico Gómez, Mexico City 06720, Mexico; jvilchisgil@gmail.com; 2 Facultad de Medicina, Universidad Nacional Autónoma de México, Mexico City 04510, Mexico; 3 Departamento de Matemáticas, Facultad de Ciencias, Universidad Nacional Autónoma de México, Mexico City 04510, Mexico; maugomez@ciencias.unam.mx; 4 Departamento de Neonatología, Hospital Infantil de México Federico Gómez, Mexico City 06720, Mexico; dra.santillan03@gmail.com; 5 Departamento de Cardiología, Hospital Infantil de México Federico Gómez, Mexico City 06720, Mexico; begstanford12@gmail.com (B.S.-S.); inavarro2000@gmail.com (I.N.-C.); 6 Hospital Militar de Especialidades de la Mujer y Neonatología, Secretaría de la Defensa Nacional, Mexico City 11200, Mexico; drajackiegomezlopez@yahoo.com.mx; 7 Laboratorio de Investigación en Patogénesis Molecular, Hospital Infantil de México Federico Gómez, Mexico City 06720, Mexico; mcme08@gmail.com (M.C.M.-E.); javiertgranados@gmail.com (J.T.G.-R.); 8 Laboratorio de Investigación en Cardiogenética, Hospital Infantil de México Federico Gómez, Mexico City 06720, Mexico; 9 Escuela Superior de Medicina, Instituto Politécnico Nacional, Mexico City 11340, Mexico

**Keywords:** congenital heart defects, B-complex vitamins, maternal nutrition, pregnancy, one-carbon metabolism, principal component analysis

## Abstract

Background/Objectives: Congenital heart defects (CHDs) are among the most common congenital anomalies worldwide, and maternal nutrition during early pregnancy may influence their development. Although previous studies have mainly evaluated individual B-complex vitamins, the contribution of their combined dietary intake to CHD risk remains poorly understood. This study investigated the association between maternal dietary B-complex vitamin intake and the risk of complex CHDs in a Mexican case–control study. Methods: Maternal dietary intake during pregnancy was assessed using a validated food frequency questionnaire, and nutrient intakes were energy-adjusted. Principal component analysis was used to construct a B-Complex Index based on the combined intake of vitamins B1, B2, B3, B6, and B12. Associations were evaluated using multivariable logistic regression adjusted for maternal age, total energy intake, multivitamin supplementation during pregnancy, and periconceptional folic acid supplementation. Results: Mothers of children with complex CHDs had significantly lower dietary intakes of B-complex vitamins than control mothers. The B-Complex Index was independently associated with lower odds of complex CHDs (adjusted OR = 0.707, 95% CI: 0.586–0.843; *p* = 0.0001). Conclusions: These findings suggest that evaluating the overall maternal dietary pattern of selected B-complex vitamins may provide complementary information regarding the nutritional environment relevant to early cardiac development. Prospective studies integrating dietary assessment, biomarkers, and genetic susceptibility are warranted to confirm these findings and further elucidate the underlying biological mechanisms.

## 1. Introduction

Congenital heart defects (CHDs) are a heterogeneous group of structural abnormalities of the heart and/or great vessels that arise during embryonic development; they affect 3 to 8 out of every 1000 live births [1], and in Mexico, it is estimated that 12,000 to 16,000 children are born each year with some form of CHDs [2]. Despite major advances in prenatal diagnosis, surgical treatment, and long-term clinical care, the etiology of CHDs remains incompletely understood. Current evidence supports a multifactorial origin in which genetic susceptibility interacts with maternal and environmental factors during critical stages of embryonic cardiac development [3,4,5,6,7].

Maternal environmental factors associated with CHDs include infections during pregnancy, exposure to teratogenic medications, chemical agents, and psychoactive substances, as well as maternal disorders such as diabetes mellitus, obesity, preeclampsia, epilepsy, and phenylketonuria [3,4,5,6,7,8]. Maternal age, parity, interpregnancy interval, and nutritional status have also been associated [9,10,11,12]. Because cardiac morphogenesis occurs during the first weeks of gestation, maternal nutrition before and during early pregnancy plays a pivotal role in shaping the intrauterine environment by providing essential macro- and micronutrients required for cellular proliferation, differentiation, energy metabolism, and epigenetic regulation [13,14,15,16].

Among dietary factors, B-complex vitamins are a group of metabolically interconnected micronutrients that function as essential cofactors in numerous biochemical pathways necessary for normal embryonic development [17]. Thiamin (B1), riboflavin (B2), niacin (B3), pyridoxine (B6), and cobalamin (B12) facilitate cellular energy production, mitochondrial function [18], amino acid metabolism, nucleotide synthesis, and redox homeostasis. Moreover, riboflavin, pyridoxine, and cobalamin are particularly involved in regulating one-carbon metabolism and homocysteine homeostasis [13,17,19,20]. These coordinated metabolic processes are vital for maintaining the rapid cellular proliferation, differentiation, and tissue remodeling characteristic of organogenesis [20,21]. Therefore, inadequate maternal intake of one or more B-complex vitamins may compromise these interconnected pathways, potentially impairing embryonic development through various biological mechanisms, including oxidative stress, altered methylation, and defective cellular metabolism [14,22,23,24,25].

Considering these complementary biological functions, epidemiological studies have investigated whether maternal consumption of selected B vitamins influences the risk of congenital anomalies. Previous epidemiological research has primarily examined associations between maternal intake of folate, vitamin B6, and vitamin B12 and the occurrence of congenital anomalies, including CHDs. The existing evidence has predominantly concentrated on these vitamins due to their roles in one-carbon metabolism [22,23,26,27]. Nevertheless, these vitamins participate in a broader network of interconnected metabolic pathways, encompassing energy metabolism, mitochondrial function, oxidative stress regulation, and cellular proliferation. This suggests that the overall dietary profile of selected B-complex vitamins may provide complementary information regarding maternal nutritional status during embryogenesis, in addition to analyses based on individual vitamin intakes [17,18,19,20]. Additionally, data on maternal B-complex vitamin intake and CHDs in Latin American populations remain limited, despite the unique nutritional characteristics and genetic backgrounds of these populations.

Therefore, the present study aimed to evaluate the association between maternal dietary intake of selected B-complex vitamins (summarized as a dietary B-complex vitamin index), folic acid intake, and folic acid supplementation and the risk of complex CHDs in a Mexican population.

## 2. Materials and Methods

### 2.1. Subjects

After written informed consent was obtained, consecutive mother–child pairs diagnosed with complex CHDs were recruited between January 2024 and December 2025. CHDs were defined as congenital cardiac malformations that, due to their anatomical and/or pathophysiological characteristics, are associated with the highest lifetime risk of morbidity and mortality and require specialized medical care [28]. These CHDs were identified through clinical evaluation and subsequently confirmed by echocardiography. CHDs were classified as “isolated” if they were not associated with additional congenital anomalies. The control group was selected consecutively from an existing maternal nutrition database established in 2019 and comprised healthy mother–infant pairs, with infants aged 28 days or younger, no significant perinatal history, no adverse birth events, and normal cardiopulmonary adaptation. Controls were selected using the same eligibility criteria, except for the absence of CHDs. In total, 75 mothers of children with complex CHDs and 160 control mothers participated in this case–control study. Exclusion criteria included women with pre-existing or gestational type 1 or type 2 diabetes, as well as those with a history of exposure to teratogens—including smoking, alcoholism, substance abuse, and use of medications such as barbiturates, lithium, methotrexate, chemotherapy, or retinoic acid—that could predispose to CHDs. Additionally, women with positive TORCH test results were excluded. A comprehensive perinatal medical history was documented, a physical examination was performed, and the women’s height was measured with a stadiometer (SECA^®^, Hamburg, Germany) at enrollment. At birth, anthropometric data for newborns—including weight, length, gestational age, mode of delivery (vaginal or cesarean), sex, and Apgar and Silverman–Anderson scores—were collected.

### 2.2. Frequency of Food Consumption

A food-frequency questionnaire to assess habitual dietary intake throughout pregnancy was administered by a trained physician to all participants in the initial postpartum weeks, including both the case and control groups. The questionnaire has been validated to estimate folate intake in the Mexican population [29]. Before completing the questionnaire, participants were instructed to report on their food consumption throughout pregnancy. In the case group, the questionnaire was administered within six months after delivery, as interviews were limited to this period to minimize the potential for recall bias. Food consumption was quantified using units of measurement (pieces, cups, plates, or spoons) and their respective sizes (small, medium, or large). Consumption frequency was converted to grams or milliliters per day. Participants’ daily intake of energy (calories), macronutrients (carbohydrates, fats, and proteins), and vitamins (B1, B2, B3, B6, B9, and B12) was calculated using the Food Processor SQL software (version 10.9.0, 2011; ESHA Research, Salem, OR, USA) and Mexican food composition tables, which include data on traditional Mexican foods. Therefore, nutrient estimates were derived from the foods reported in the FFQ. Information on dietary supplements other than folic acid was not collected as part of the study protocol, and only folic acid supplementation was assessed separately as an independent variable.

### 2.3. Dietary Data Transformation and Energy Adjustment

To rigorously control the confounding effect of total energy intake, which inherently influences absolute consumption levels, dietary variables were standardized by nutrients to ensure comparability prior to inferential analysis. Micronutrients, including the primary variables of interest—B-complex vitamins (thiamine, riboflavin, niacin, pyridoxine, and cobalamin) and folates—along with whole food groups, were energy-adjusted using the standard nutrient density method, expressed as amounts per 1000 kcal. Macronutrients (carbohydrates, proteins, and fats) were standardized to represent their percentages of total daily energy contribution using appropriate Atwater conversion factors (4 kcal/g for carbohydrates and proteins and 9 kcal/g for fats). Although extensive data curation covered the full dietary spectrum, subsequent multivariate models were a priori focused strictly on the B-complex vitamins to evaluate their combined dietary pattern in relation to complex CHDs.

### 2.4. Principal Component Analysis and Index Construction

To address the high multicollinearity inherent in maternal dietary micronutrient data and to capture the combined dietary pattern of the selected B-complex vitamins, Principal Component Analysis (PCA) was performed on the energy-adjusted densities of vitamins B1, B2, B3, B6, and B12. Before extraction, the nutrient density variables were standardized to Z-scores to ensure that differences in measurement scales (mg versus μg) did not disproportionately bias the total variance. The first principal component (PC1) was retained because it explained the greatest proportion of the total variance, indicating robust informational convergence among the individual B vitamins. Individual factor scores from this first dimension were extracted for each participant to serve as a continuous, orthogonal, and energy-independent metric, hereafter formally designated as the “B-Complex Index.” This composite index was used in subsequent multivariate analyses to ensure statistical stability and eliminate the risk of variance inflation. Dietary folate intake was intentionally not included in the PCA because folate intake and periconceptional folic acid supplementation were prespecified exposures of interest and were evaluated separately in the statistical analyses. Accordingly, the selected B-Complex Index was specifically designed to summarize the combined dietary intake pattern of vitamins B1, B2, B3, B6, and B12 rather than incorporate all B vitamins into a single composite measure. The B-Complex Index was defined as the score of the first principal component (PC1), representing a weighted combination of the standardized dietary intakes of the selected B-complex vitamins (B1, B2, B3, B6, and B12) based on their PCA loadings.

### 2.5. Statistical Analysis

Descriptive statistics for continuous variables, including demographic characteristics and standardized dietary intakes, were summarized using medians and their corresponding first and third quartiles (Q1–Q3) because nutritional data are typically non-normal.

To compare continuous variables between the case and control groups, a rigorous rank-based approach was used. First, the assumption of homoscedasticity (homogeneity of variances) was evaluated for each variable using the Fligner–Killeen test, which is robust to non-normal distributions.

Subsequent hypothesis tests were selected according to the variance structure of each variable. For distributions exhibiting homogeneous variances (*p* ≥ 0.05) in the Fligner–Killeen test, group comparisons were performed using the Wilcoxon rank-sum test (Mann–Whitney U test) [30]. Conversely, for variables demonstrating significant heteroscedasticity (*p* < 0.05), the Brunner–Munzel test was utilized [31]. This conditional approach ensures robust statistical inference by preventing the inflation of Type I errors that occur when standard non-parametric tests are applied to heteroscedastic data. All statistical tests were two-sided, and statistical significance was defined at an alpha level of 0.05.

To assess the independent association between the maternal B-Complex Index and the likelihood of congenital defects, a multivariable logistic regression model was constructed [32]. The model was adjusted for clinically relevant covariates, including maternal age, total caloric intake, multivitamin supplementation during pregnancy, and preconception folic acid intake. To rigorously evaluate the assumptions of the logistic model, simulated randomized quantile residuals were generated using the DHARMa framework [33]. Goodness-of-fit was assessed using the Kolmogorov–Smirnov, dispersion, and outlier tests on the simulated residuals, which showed no evidence of model misspecification or overdispersion. Influential observations were further examined using Cook’s distance and leverage plots; no data points exceeded the conventional threshold for undue influence (Cook’s D < 0.5), supporting the stability of the fitted model. The corresponding diagnostic plots are included in Supplementary Figure S1.

All data management, transformations, and statistical analyses were conducted utilizing the R statistical computing environment (version 4.5.1) [34]. Data curation and scaling procedures were executed employing the dplyr (version 1.2.1) and tidyr (version 1.3.1) packages. For baseline comparisons, conditional hypothesis testing—specifically, the Brunner–Munzel test for heteroscedastic data—was performed using the lawstat package (version 3.6) [35]. Dimensionality reduction via Principal Component Analysis (PCA) and the corresponding spatial biplots were generated utilizing factoextra (version 1.0.7) [36], while nutrient density boxplots were produced with ggplot2 (version 3.5.2) [37]. Lastly, goodness-of-fit and assumptions of the multivariable logistic regression model were meticulously performed using simulated randomized quantile residuals within the DHARMa framework (version 0.4.6) [33].

## 3. Results

### 3.1. Distribution of Complex Congenital Heart Defects

A total of 75 children with complex congenital heart defects (CHDs) were included in the study. The most frequent diagnoses were total anomalous pulmonary venous connection (13/75, 17.3%) and tetralogy of Fallot (13/75, 17.3%), followed by coarctation of the aorta (7/75, 9.3%), transposition of the great arteries (7/75, 9.3%), pulmonary atresia without ventricular septal defect (6/75, 8.0%), and coarctation of the aorta with ventricular septal defect (4/75, 5.3%). The remaining complex CHD diagnoses accounted for fewer than 5% of cases.

### 3.2. Maternal and Neonatal Characteristics

Maternal and neonatal characteristics of cases and controls are summarized in Table 1. Mothers of infants with complex CHDs were significantly older than those in the control group (*p* = 0.002). No significant differences were observed between the groups in pre-pregnancy body mass index or periconceptional folic acid supplementation. However, vitamin supplementation during pregnancy was less common among case mothers than among controls (*p* = 0.022).

Among neonatal characteristics, infants with complex CHDs had a significantly lower birth weight compared to controls (*p* = 0.001). Additionally, cesarean delivery was more prevalent among cases than controls (*p* < 0.001). No significant differences were observed in birth length, gestational age, infant sex, or the 1-min Apgar score.

### 3.3. Maternal Dietary Intake During Pregnancy

Maternal dietary intake during pregnancy is presented in Table 2. Total energy intake showed a trend toward a difference between case and control mothers, but this association did not reach statistical significance (*p* = 0.054). Compared with controls, mothers of infants with complex CHDs had lower protein (*p* = 0.009) and lipid (*p* = 0.007) intakes, whereas carbohydrate and folate intakes were comparable between groups (*p* > 0.05).

Mothers of infants diagnosed with complex CHD had markedly lower dietary intakes of all evaluated B-complex vitamins, including thiamine (vitamin B1), riboflavin (vitamin B2), niacin (vitamin B3), pyridoxine (vitamin B6), and cobalamin (vitamin B12), compared with control mothers (all *p* < 0.01).

### 3.4. Energy-Adjusted Dietary Intake of B-Complex Vitamins

**Figure 1:**
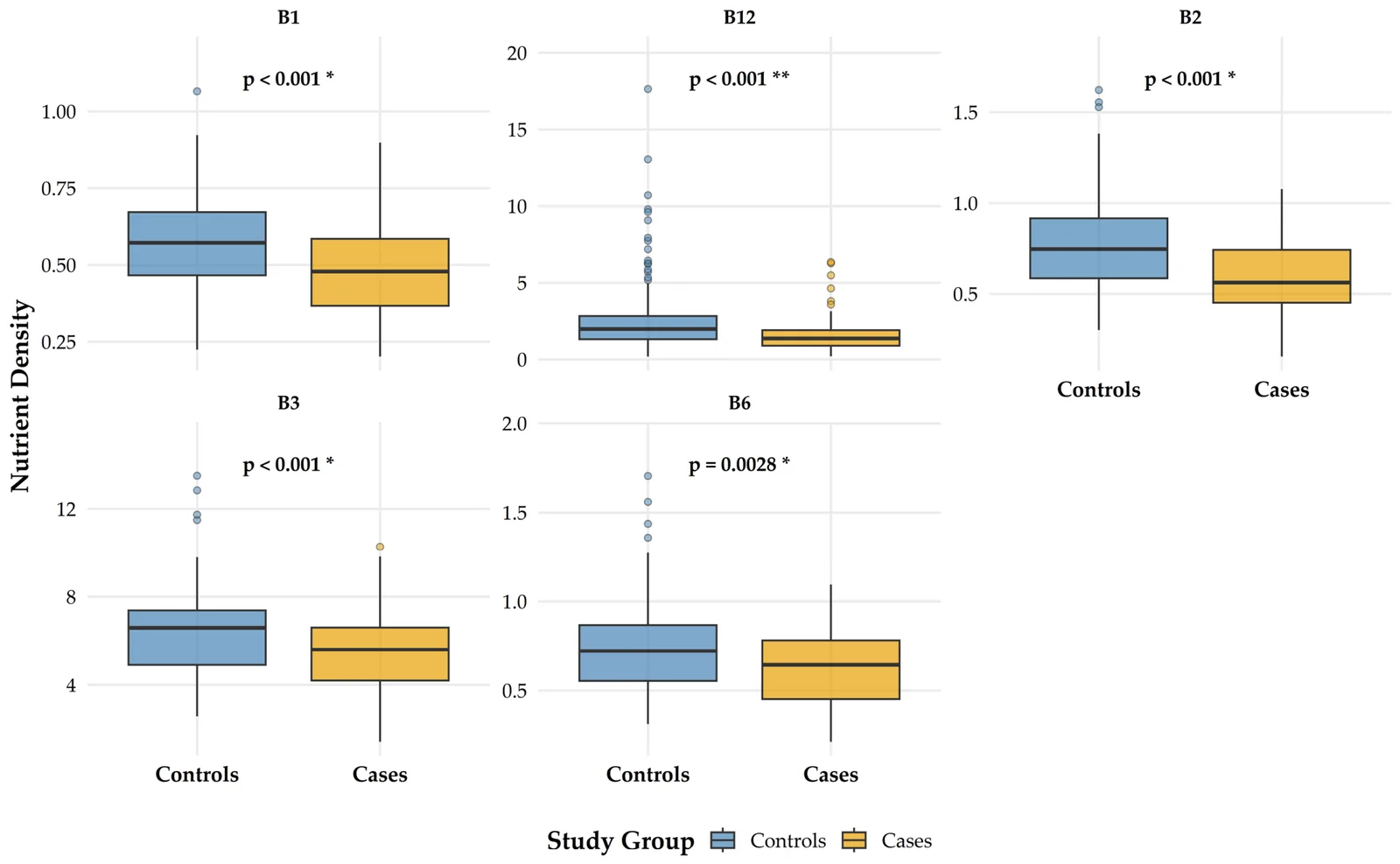
Energy-adjusted dietary density of B-complex vitamins in the maternal diet, stratified by study group. Boxplots show the median and first and third quartiles (Q1–Q3) of nutrient densities, standardized per 1000 kcal, for the Control (blue) and Case (orange) groups. Outliers are shown as semi-transparent points. Hypothesis testing was assigned conditionally based on variance homogeneity, previously evaluated via the Fligner–Killeen test: single asterisks (*) denote statistical significance computed via the Wilcoxon rank-sum test for homoscedastic distributions, whereas double asterisks (**) denote statistical significance computed via the Brunner–Munzel test for heteroscedastic distributions (e.g., Vitamin B12). Across all evaluated B-complex dimensions, the maternal diet in the case group exhibited significantly lower nutrient density than that of controls.

Since dietary intake is affected by total energy consumption, B-complex vitamin intakes were adjusted to a standard of 1000 kcal to evaluate nutrient density independently from overall caloric intake (Figure 1). Mothers of infants with complex CHD exhibited significantly lower energy-adjusted intakes of thiamine (vitamin B1), riboflavin (vitamin B2), niacin (vitamin B3), pyridoxine (vitamin B6), and cobalamin (vitamin B12) compared to control mothers (all *p* < 0.01). Considering the consistent pattern observed across the assessed B-complex vitamins, principal component analysis was subsequently conducted to establish an integrated dietary B-complex vitamin index.

### 3.5. Multivariate Dietary Patterns and the B-Complex Index

**Figure 2:**
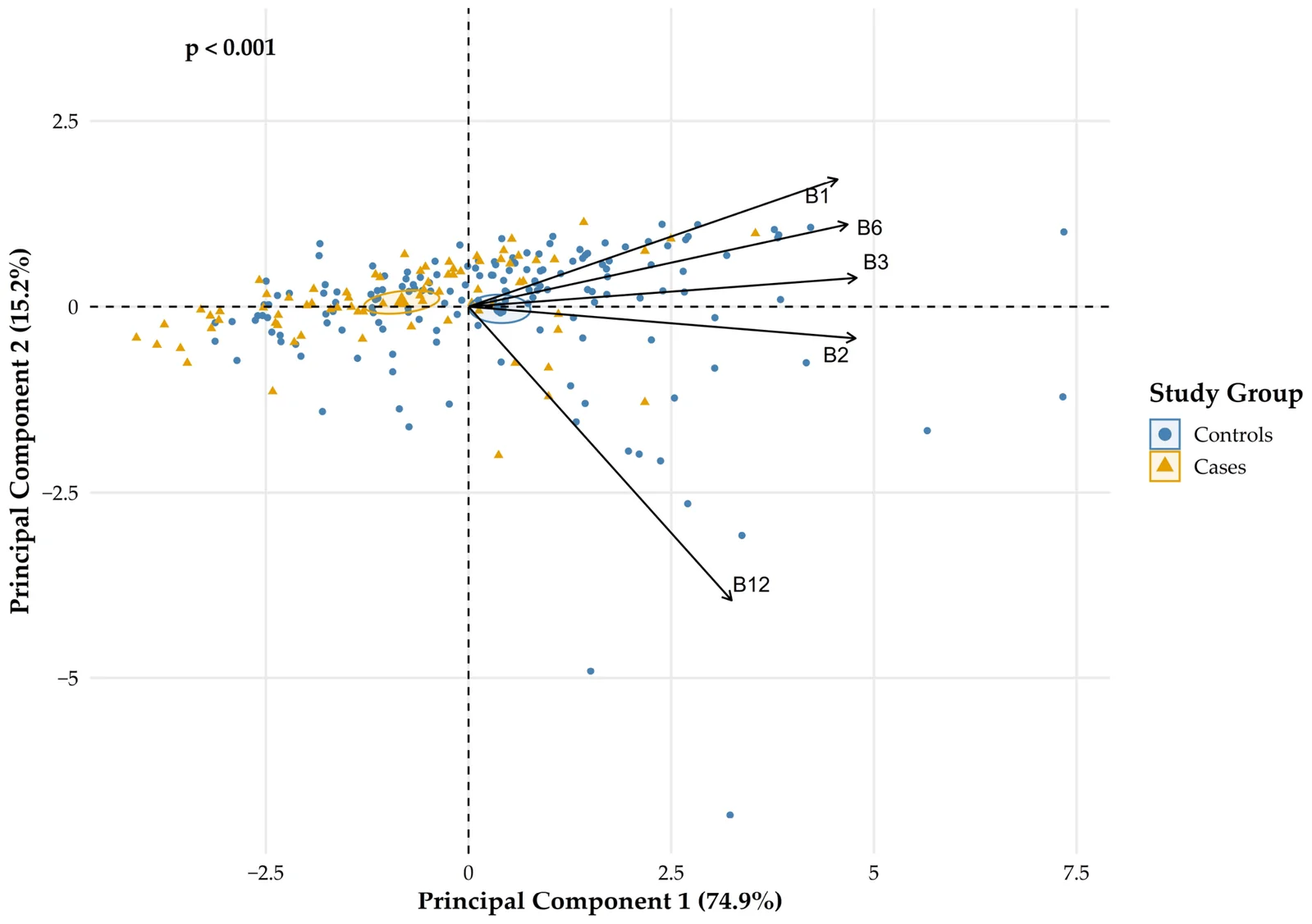
Illustrates a Principal Component Analysis (PCA) biplot depicting energy-adjusted maternal B-complex vitamin densities. The biplot demonstrates the multivariate distribution of the study population and the loading vectors of individual B vitamins along the first two principal components. Each patient is represented by a score, with color-coding indicating study group membership (Controls in blue, Cases in orange), accompanied by corresponding 95% confidence ellipses. Directional vectors (black arrows) denote the loadings of the five evaluated B vitamins (B1, B2, B3, B6, and B12). The horizontal axis (PC1) accounts for 74.9% of the total variance, representing the overall density of the B-complex in the maternal diet and serving as the basis for the B-Complex Index. The annotation in the upper-left corner displays the statistical significance (*p*-value) of the mean index difference between groups, calculated using an independent-samples *t*-test.

To evaluate the integrated dietary density of the B-complex vitamins, a Principal Component Analysis (PCA) was conducted. The spatial distribution of the study population and the behavior of the nutrient vectors are shown in the PCA biplot (Figure 2). The first principal component (PC1), defining the B-Complex Index, captured 74.9% of the total dietary variance. Notably, all five vitamin vectors (B1, B2, B3, B6, and B12) loaded heavily and positively along this primary axis, confirming a strong pattern of correlated intake.

The PCA biplot demonstrated a distinct separation between the study groups along PC1. The 95% confidence ellipses, representing the multivariate centroids of each group, indicate clear spatial segregation; the centroid of the control group is positioned toward the positive end of the PC1 axis (suggesting higher overall B-complex density), whereas the centroid of the case group is shifted toward the negative end. To quantitatively assess this visual disparity, an independent-samples *t*-test was conducted on the B-Complex Index. The results confirmed a highly significant difference in the mean B-Complex Index between the groups (*p* < 0.001), indicating that the maternal diet in the case group was consistently and comprehensively lower in B-complex vitamin intake than in the control group.

### 3.6. Multivariable Logistic Regression Analysis

The multivariable logistic regression model demonstrated a highly significant protective association between the maternal B-Complex Index and the outcome (see Table 3). After adjusting for maternal age, total energy intake, and supplementation practices, the B-Complex Index persisted as a robust independent predictor. Specifically, each one-unit increase in the B-Complex Index correlated with a 29.3% decrease in the likelihood of having a child with complex CHDs (adjusted OR = 0.707; 95% CI: 0.586–0.843; *p* = 0.0001).

Among the evaluated covariates, maternal age showed a modest positive association with CHD risk (adjusted OR = 1.058; 95% CI: 1.007–1.112; *p* = 0.0248). Periconceptional folic acid supplementation was independently associated with lower odds of complex CHDs (adjusted OR = 0.337; 95% CI: 0.090–0.996; *p* = 0.0491), whereas vitamin supplementation during pregnancy was not significantly associated with the outcome (*p* = 0.1049).

## 4. Discussion

In this study, we found that a higher maternal dietary B-complex vitamin index was associated with a lower risk of complex CHDs in offspring, even after accounting for maternal age, total energy intake, vitamin supplementation during pregnancy, and periconceptional folic acid supplementation. Rather than evaluating each vitamin B separately, we assessed the combined dietary intake as an integrated nutritional pattern. This approach reflects the biological interactions among selected B-complex vitamins, which participate in interconnected metabolic pathways involved in one-carbon metabolism, mitochondrial energy production, amino acid metabolism, and cellular proliferation, all of which are essential for normal cardiac development during early embryogenesis [17,18,19,20,21]. Therefore, evaluating these vitamins as a dietary pattern may provide complementary information regarding the maternal nutritional environment, in addition to analyses based on individual vitamin intakes [17].

Selected B-complex vitamins participate in several interconnected metabolic pathways essential for normal cardiac morphogenesis. Vitamins B6 and B12 contribute to one-carbon and homocysteine metabolism [13,15,17,20], whereas vitamins B1, B2, and B3 function as essential cofactors in mitochondrial energy production and cellular oxidation-reduction reactions [18,19,25]. Together, these vitamins also support amino acid metabolism [15,17] and cellular processes required for rapid proliferation and differentiation during early embryogenesis [16,17,20]. B-complex vitamins involved in one-carbon metabolism contribute to methyl-group metabolism and DNA methylation, an essential epigenetic mechanism that precisely regulates gene expression during cardiac development [15,16,20]. Consequently, inadequate intake of several B-complex vitamins, even in the absence of a severe deficiency of any single vitamin, may disrupt these interconnected pathways, alter the coordinated regulation of developmental genes, and ultimately contribute to abnormal cardiac morphogenesis [14,22,23,24,25].

Previous epidemiological studies have examined individual B-complex vitamins, specifically folate, vitamin B6, and vitamin B12, in relation to congenital anomalies and CHDs, with inconsistent results across different populations [26,38,39,40]. Overall, these studies indicate that sufficient maternal intake of these vitamins is associated with a reduced risk of CHDs, attributable to their functions in one-carbon metabolism and homocysteine homeostasis [13,17,22,23,26,38,39]. Nonetheless, most research has considered these nutrients separately, despite their interconnected metabolic roles. Our findings suggest that evaluating selected B-complex vitamins as an integrated dietary pattern provides a complementary approach for characterizing the maternal nutritional environment during pregnancy. Because these vitamins participate in interconnected metabolic pathways involved in embryonic development, assessing them jointly may offer additional insight into their combined association with the risk of complex CHDs. In addition to differences in the intake of selected B-complex vitamins, maternal protein and lipid intake also differed between the case and control groups. These findings may reflect broader differences in overall dietary composition, particularly in the consumption of foods that are important dietary sources of several selected B-complex vitamins, rather than isolated variations in specific nutrients. However, because protein and lipid intake were not the primary focus of this study, their independent contribution to the risk of complex CHDs could not be determined and should be explored in future studies.

The use of the dietary-selected B-complex vitamin index represents a complementary approach for assessing maternal nutritional status during pregnancy. Given that these vitamins participate in interconnected metabolic pathways, evaluating them as an integrated dietary pattern may provide complementary information regarding maternal nutritional intake that may not be fully captured by analyses of individual vitamins. Principal component analysis allowed us to summarize the shared variability among selected B-complex vitamins into a single dietary index, thereby mitigating the effects of collinearity and providing an integrated measure of the combined dietary intake pattern of selected B-complex vitamins. This integrated approach enabled assessment of the joint dietary intake of selected B-complex vitamins as a single composite measure. In the multivariable analysis, the B-Complex Index remained independently associated with complex CHDs after adjustment for potential confounding variables.

Accumulating evidence suggests that both maternal nutritional status and genetic susceptibility play contributory roles in the risk of CHDs through shared metabolic pathways. Recent findings further corroborate the notion that maternal folate availability and genetic variation within one-carbon metabolism pathways jointly influence susceptibility to CHDs, including variants in *MTHFD1*, *MTHFD2*, *MTRR*, and other genes [41,42,43]. Likewise, research conducted within Mexican populations has identified associations between *MTHFR* variants and CHDs, thereby supporting the biological significance of one-carbon metabolism during cardiac embryogenesis [44,45]. Although this study did not assess genetic variants, these previous findings provide a biologically plausible context for interpreting the observed association between the maternal dietary B-Complex Index and the risk of complex CHDs.

### Strengths and Limitations

The present study has several strengths. First, maternal dietary intake was assessed with a validated food frequency questionnaire, allowing evaluation of habitual dietary intake throughout pregnancy. Second, principal component analysis was used to construct an integrated dietary B-Complex Index based on selected B-complex vitamins, providing a composite measure of their combined dietary intake pattern while accounting for intercorrelations among these micronutrients. Third, this study provides evidence from a Mexican population, which remains underrepresented in studies evaluating maternal nutrition and CHDs.

Nevertheless, several limitations should be acknowledged. Because of the case–control design, causal relationships cannot be established, and dietary information may be subject to recall bias. In addition, dietary intake was estimated using a food-frequency questionnaire rather than circulating biomarkers; therefore, nutrient intake may not fully reflect maternal vitamin status during early pregnancy. Although the food-frequency questionnaire used in this study was specifically validated for folate intake in the Mexican population, food-frequency questionnaires are widely accepted and extensively used in nutritional epidemiology to estimate habitual intake of multiple nutrients, including B-complex vitamins. However, because a specific validation for vitamins B1, B2, B3, B6, and B12 has not been reported in this population, these estimates should be interpreted with appropriate caution. Furthermore, the FFQ assessed habitual dietary intake throughout pregnancy and did not specifically distinguish intake during the critical period of cardiac organogenesis. Maternal dietary patterns may also change across preconception, pregnancy, and postpartum/lactation periods. Consequently, dietary information collected after delivery may not fully reflect maternal nutritional intake during the critical stages of embryonic cardiac development. Although the same eligibility criteria, validated food-frequency questionnaire, and standardized data collection procedures were applied to both groups, controls were recruited from a different healthcare institution than cases. Therefore, unmeasured differences in socioeconomic characteristics or healthcare access between the two populations cannot be completely ruled out. Although the multivariable model adjusted for maternal age, total energy intake, and supplementation practices, residual confounding by other maternal or lifestyle-related factors cannot be completely ruled out, such as the socioeconomic level or educational level of the mother or family. Additionally, the sample size was determined by the number of consecutive eligible participants available during the study period, which may have limited the statistical power and precision of some estimates. Therefore, the findings should be interpreted with appropriate caution and confirmed in prospective studies with repeated dietary assessments and objective nutritional biomarkers.

## 5. Conclusions

Our findings suggest that a greater maternal dietary intake of selected B-complex vitamins, evaluated as an integrated dietary pattern, is associated with a lower risk of complex CHDs. These findings highlight the importance of considering the collective metabolic contributions of selected B-complex vitamins during the early stages of cardiac development and suggest that evaluating these vitamins as a dietary pattern may provide complementary information to analyses based on individual vitamin intakes. Future prospective studies integrating dietary assessment, biomarkers, and genetic susceptibility are warranted to confirm these findings and further elucidate the underlying biological mechanisms.

## Figures and Tables

**Table 1:** Clinical and Perinatal Characteristics of Mothers and Newborns.

	Case Group Median (Q1, Q3) (*n* = 75)	Control Group Median (Q1, Q3) (*n* = 160)	Fligner–Killeen Test *p* *	*p* ^†^
Mothers				
**Age** (y)	24.0 (21.0, 29.0)	22.0 (19.0, 25.2)	0.342	0.002
**BMI before pregnancy** (kg/m^2^)	23.7 (21.7, 27.9)	23.4 (21.1, 26.5)	0.190	0.248
**Perinatal folic acid supplementation,** n (%)	73 (97.3)	149 (93.1)	NA	0.234
**Vitamin supplementation during pregnancy,** n (%)	56 (74.7)	140 (87.5)	NA	0.022
Newborns				
**Weight** (kg)	2850 (2495, 3220)	3105 (2877, 3322)	0.001	0.001
**Length** (cm)	49 (47, 51)	49.3 (48, 50)	0.002	0.573
**Sex, male,** n (%)	40 (53.3)	82 (51.3)	NA	0.781 ^†^
**Weeks of gestation**	39 (38, 40)	39 (38, 40)	0.165	0.742
**Cesarean delivery,** n (%)	70 (93.3)	49 (30.6)	NA	<0.001 ^†^
**Apgar score at 1 min (≤8),** n (%)	66 (88.0)	139 (86.8)	NA	0.999 ^†^

* Fligner Killeen Test. ^†^ Brunner–Munzel Test, Wilcoxon Test or Fisher Test. BMI, Body Mass Index.

**Table 2:** Maternal dietary intake of energy, macronutrients, and B-complex vitamins during pregnancy.

Energy-Adjusted Daily Nutrient Intake 1000 kcal	Case Group Median (Q1, Q3) (*n* = 75)	Control Group Median (Q1, Q3) (*n* = 160)	*p* ^†^
**Energy**, (kcal)	2752 (2106, 3630)	2505 (1941, 3350)	0.054
**Protein** (g)	38.3 (34.9, 42.7)	40.4 (36.9, 42.7)	0.009
**Carbohydrates** (g)	59.1 (52.7, 64.1)	56.9 (51.9, 64.8)	0.474
**Lipids** (g)	25.8 (22.3, 28.8)	27.0 (24.4, 29.7)	0.007
**Folate** (μg)	147.2 (98.8, 183.7)	151.5 (119.5, 184.8)	0.390
**Vitamin B1** (mg)	0.48 (0.37, 0.59)	0.57 (0.47, 0.67)	<0.001
**Vitamin B2** (mg)	0.56 (0.45, 0.74)	0.75 (0.59, 0.92)	0.001
**Vitamin B3** (mg)	5.6 (4.21, 6.59)	6.58 (4.91, 7.38)	<0.001
**Vitamin B6** (mg)	0.72 (0.56, 0.87)	0.64 (0.45, 0.78)	0.003
**Vitamin B12** (μg)	1.37 (0.89, 1.91)	1.98 (1.32, 2.83)	<0.001

Values are presented as median (Q1–Q3). Macronutrient and micronutrient intakes were energy-adjusted and expressed per 1000 kcal. ^†^ *p*-values were derived from the Wilcoxon rank-sum test for homoscedastic data or the Brunner–Munzel test for heteroscedastic data. The assumption of equal variances was previously evaluated for each variable using the Fligner–Killeen test.

**Table 3:** Multivariable logistic regression analysis of factors associated with complex congenital heart defects.

Variable	Adjusted OR	CI 95%	*p*
**B-complex vitamin index**	0.707	(0.586–0.843)	0.0001
**Maternal age (years)**	1.058	(1.007–1.112)	0.0248
**Total energy intake**	1.000	(1.000–1.001)	0.0331
**Vitamin supplementation**	0.523	(0.239–1.146)	0.1049
**Periconceptional folic acid supplementation**	0.337	(0.090–0.996)	0.0491

OR: Odds Ratio; CI 95%: 95% confidence interval.

## Data Availability

The data presented in this study are available upon request from the corresponding author due to ethical and privacy restrictions to protect participant confidentiality. The data are not publicly available because they contain information that could compromise the privacy of research participants.

## Supplementary Materials

The following supporting information can be downloaded at: https://www.mdpi.com/article/10.3390/nu18162693/s1, Figure S1. Diagnostic plots for the multivariable logistic regression model, including DHARMa residual diagnostics, Cook’s distance, and residuals versus leverage plots.
